# Progress in Opthalmology

**Published:** 1903-10-24

**Authors:** 


### Progress in Ophthalmology.

**Ligation of the Canaliculi in Treatment of Suppuration of the Cornea.**—It is well known that one of the dangers after cataract operation results from pus regurgitating back through the canaliculi from the lachrymal sac where there is chronic suppuration of its mucous membrane, and one has seen several times the suppurating sac excised for the purpose of avoiding this source of infection. In Vienna Professor Fuchs[1] under these circumstances used to cut into the sac through the skin, and having plugged it with iodoform gauze, at once proceeded to the cataract operation. To obviate this entire destruction of the sac, Buller, of Montreal, advocates mere ligature of the canaliculi. Having had a successful case of cataract extraction where this manœuvre was carried out, he tried it in a case of extensive suppurative keratitis complicated by disease of the lachrymal sac. In this case the sac was syringed out with a 1—3,000 solution of hydr. perchlor., and the upper and lower canaliculi were then ligatured. The conjunctiva was thoroughly cleansed and the diseased cornea touched with formalin solution, the conjunctival sac filled with a 10 per cent. Airol ointment, and a compress bandage applied. For two days there was some reaction from the formalin, but after that a steady improvement. After the corneal trouble was well the author states he had no trouble in reopening the canaliculi, nor was there any worry from the increased accumulation in the tear sac.

**Peritomy for Diffuse Corneitis and other Affections of the Cornea.**—The operation of peritomy, which at first was performed for pannus in cases of trachoma, was soon given up as both useless and beginning at the wrong end of the trouble. The pannus, being the result of the rough granulation tissue rubbing on the cornea, could not be cured unless the trachoma was removed, or at least improved. Nowadays surgeons begin with the cure, or improvement of the trachoma, and this being accomplished, the pannus in a large measure cures itself. Having failed in producing a cure of pannus, the operation went entirely out of use; but there is no doubt of its efficacy in other conditions of the cornea, and Snell[2] recommends it very strongly in diffuse corneitis and those corneal ulcers where there is a leash of vessels extending from the corneal margin to the ulcer, and which are so liable to relapse. He says:—The operation is a brief one, and if cocaine is freely instilled it is usually sufficient. In addition, however, he sometimes uses adrenalin, instilling it two or three times before commencing the operation. This affords the very great advantage of allowing the operation to be a bloodless one, and also the adrenalin seems to increase the anæsthetic effect of the cocaine. A fold of conjunctiva just beyond the cornea at the upper part is seized with forceps and then snipped with curved scissors; from this point the conjunctiva is severed all round the cornea at a distance of 2 to 3 mm. The portion adhering to the cornea is then dissected off the sclerotic and removed. The division of the conjunctiva is facilitated by using a pair of scissors having one blade somewhat longer than the other and ending with a bulbous point which readily runs under the conjunctiva. Recovery is usually quick, especially in young subjects. For a few days a rim of bare sclerotic is visible, but after a very short time there is little indication of any operation having been performed. In a few cases he performed a partial peritomy opposite any particular portion of the cornea that it was specially desired to affect. He has done peritomy in over 100 cases of diffuse corneitis, and his experience shows it to be a remedy of great value. The beneficial effects are often quickly seen, especially in those cases which soon show the characteristic salmon patches at one border of the cornea. For a case in which the salmon patch showed at the upper part he did a partial peritomy above. The effect was good; but when a similar inflammation began in the other eye he did a complete peritomy with greater advantage. As he has seen no bad effects from the operation he always advocates a complete and not partial peritomy. A large ulcer of the cornea to which a leash of vessels ran, was treated with the ordinary methods, including the use of the cautery, but still did not heal. A partial peritomy was done, the conjunctiva being severed over an area corresponding to one half of the cornea opposite to which the ulcer was situated. The ulcer then quickly healed. Another class of case in which he advocates peritomy is that which is described as detachment of corneal epithelium. In such an instance, which had been continuous for three months, he did a peritomy recently with marked success. In a recent case of relapsing iritis in a young man, peritomy rendered excellent service. In his opinion, therefore, we possess in peritomy a most useful means of treating diffuse corneitis, and in many other corneal affections it is a remedy of great value.

**Dislocation of Eyeball.**—To show to what length injuries may go without permanent trouble, we may refer to a case, shown by Mr. Lawford,[3] at the Ophthalmological Society, where there was complete dislocation of the eyeball in a child, aged seven, as the result of a fall against an iron fender. Reduction was easily effected under chloroform, and recovery took place without any defect of vision, and with unimpaired movements of the eyeball. Slight proptosis was noticed for a month after the accident, but no restriction of ocular movements could be detected, even three days after the reduction of the eyeball. One would have imagined that the optic nerve would have been torn, or at any rate stretched so violently as to cause some visual defect, and this case shows how much the optic nerve is regulated in its length to allow the free movements of the eyeball under normal conditions.

**The X-rays in Trachoma.**—Mayou,[4] in a paper on the treatment of trachoma, writes that the idea first occurred to him when treating lupus and rodent ulcer of the lids on finding that no serious damage was done to the globe. The histological changes in living tissues exposed to the action of $x$-rays were, a superficial irritation capable of being increased and accelerated by the simultaneous application of irritants, such as sulphate of copper. Most of the resulting leucocytosis was found around the trachoma nodules, and the cells of rodent ulcer after $x$-ray treatment of these diseases, the reason for this being that they similarly acted as irritants. It was next pointed out that with care the amount of reaction produced could be regulated and that the varying degrees of reaction might be compared with the first three degrees of burn described by Dupuytren. Cases of prolonged exposure of the globe to the $x$-rays were instanced where the only bad effects produced were the falling out of the eyelashes and conjunctivitis, and this latter trouble in the operator could be prevented by the use of lead-glass spectacles. The results of the treatment by $x$-rays were compared with those produced by copper sulphate, jequirity and other irritants, and it was pointed out that with the rays there was less destruction of tissue and subsequent cicatrization, as well as less pain. In these cases the eyelids were everted, the cornea was only exposed in severe cases where pannus was present. Owing to the infiltration set up difficulty was found in deciding when treatment should cease. Out of nine cases five remained well, one cleared up but recurred two months later, two others improved and were still under treatment, and in one case of corneal opacities following trachoma the vision had improved from perception of light to fingers at 3 feet. The advantages of this treatment were : (1) Less resultant deformity of the lid ; (2) it was painless ; (3) pannus cleared up more thoroughly. The disadvantages were : (1) All patients did not react well to $x$-rays ; (2) it was sometimes difficult to tell when treatment should cease.

[1] Scot. Med. and Surg. Jour., May 1903. [2] Lancet, May 30, 1903. [3] Brit. Med. Jour., Mar. 28, 1903. [4] Ibid.

